# An Empirical Study Comparing Unobtrusive Physiological Sensors for Stress Detection in Computer Work

**DOI:** 10.3390/s19173766

**Published:** 2019-08-30

**Authors:** Fatema Akbar, Gloria Mark, Ioannis Pavlidis, Ricardo Gutierrez-Osuna

**Affiliations:** 1 Department of Informatics, University of California, Irvine, CA 92617, USA; 2 Computational Physiology Laboratory, University of Houston, Houston, TX 77004, USA; 3 Perception, Sensing, and Instrumentation Laboratory, Texas AM University, College Station, TX 77843, USA

**Keywords:** stress, physiology, wearables, unobtrusive sensors, thermal imaging, human–computer interaction, EDA, PPG, ECG

## Abstract

Several unobtrusive sensors have been tested in studies to capture physiological reactions to stress in workplace settings. Lab studies tend to focus on assessing sensors during a specific computer task, while in situ studies tend to offer a generalized view of sensors’ efficacy for workplace stress monitoring, without discriminating different tasks. Given the variation in workplace computer activities, this study investigates the efficacy of unobtrusive sensors for stress measurement across a variety of tasks. We present a comparison of five physiological measurements obtained in a lab experiment, where participants completed six different computer tasks, while we measured their stress levels using a chest-band (ECG, respiration), a wristband (PPG and EDA), and an emerging thermal imaging method (perinasal perspiration). We found that thermal imaging can detect increased stress for most participants across all tasks, while wrist and chest sensors were less generalizable across tasks and participants. We summarize the costs and benefits of each sensor stream, and show how some computer use scenarios present usability and reliability challenges for stress monitoring with certain physiological sensors. We provide recommendations for researchers and system builders for measuring stress with physiological sensors during workplace computer use.

## 1. Introduction

Many individuals spend an increasingly significant proportion of their day at a computer, especially those in information work. Some workplace computer tasks are known to be associated with stress, such as answering emails [1,2] and presenting to a remote audience [3,4]. Besides cognitively demanding tasks, workplace stressors include time pressure [5], social pressure [6], interruptions [7] and anticipatory stress from upcoming deadlines [8,9]. Excessive exposure to workplace stress has direct effects on health and quality of life, as it can lead to burnout, diminished productivity, and several health problems including cardiovascular disease and impaired immunity functions [10,11,12]. Thus, capturing stress levels in the workplace is vital for improving our understanding of real-life stress and the factors surrounding it. Measuring stress unobtrusively and in real time at the workplace can enable affective computing applications that incorporate user’s stress and new forms of context-aware interactions [13,14]. Mental health professionals and organizational psychologists can also benefit from stress monitoring at the workplace, to better understand stress and associated factors, and to deliver interventions.

To capture stress in the workplace, several methods have been tested. Self-reported stress, also referred to as ‘perceived stress’ [15], is often considered a ground truth of stress. Several instruments have been developed, such as the Perceived Stress Scale [16], the Daily Stress Inventory [17], and one-item surveys deployed through experience sampling [18]. Although self-report instruments are commonly used in the literature, they have several limitations for stress monitoring at the workplace. Self-reports are subjective and are affected by memory and emotion expression biases. They can also be disruptive as they require the full cognitive attention of the user, and do not allow continuous stress measurement. Advances in sensor technologies embedded in wearable devices have motivated researchers to investigate the usability of unobtrusive and wearable sensors for stress measurement in the workplace [19], especially during computer use. As stress produces several physiological reactions, capturing physiological signals with sensors provides the potential to measure stress objectively, unobtrusively, and in real time.

In this paper, we review recent research on stress measurement with physiological sensors in workplace and computer use settings. We identify a gap in the literature as most studies focus on specific high-stress short-duration computer tasks to induce stress [20,21,22], which might not be representative of those in real workplace settings and can overlook issues and challenges related to stress measurement with physiological sensors during different computer activities. Workplace computer use includes activities that vary in the level of cognitive or emotional stress they could induce, the physical motions and dexterity they require, the user posture, and their duration, all which can potentially affect sensor performance. An empirical study to examine the usability and reliability of a set of unobtrusive sensors across a spectrum of computer activities is lacking.

This paper addresses the following research question: *what sensor modality functions best to measure stress across computer tasks?* To answer this research question, we compare the use of different sensor modalities across varied computer tasks, investigating the usability, reliability, and problems with each type of sensor. We report our results based on testing the sensors in a simulated office environment. The contribution of this work is as follows:

- A review of the literature on stress measurement with sensors in the workplace and laboratory studies examining computer use. Unlike reviews focusing on the results of these studies, we focus on the methods and present a summary of sensors used, tasks performed, ground truth measures, and other dependent stress variables, the number of subjects, and the duration of measurement.
- An empirical comparison of the usability and reliability of a set of sensor channels for stress response measurement during computer use, including an emerging non-contact method using thermal imaging.
- Identifying challenges for some sensors, specific to certain common computer tasks, which limit the efficacy of these sensors for continuous stress monitoring in situ in a workplace setting.
- Recommendations for researchers and system builders interested in stress measurement with unobtrusive physiological sensors during computer use.

To the best of our knowledge, this is the first study to include this collection of physiological sensor streams (heart-rate from ECG and PPG sensors, breathing rate, skin conductance and thermal imaging) which are collected simultaneously for several computer tasks, and the first study to compare thermal imaging against other wearable physiological sensors as a stress measurement technique in computer tasks (for previous studies using thermal imaging for stress detection in other contexts, see [22,23,24,25,26]).

## 2. Background

### 2.1. Physiological Stress Reactions

Stress is the result of appraising a situation as having demands exceeding resources [27] (e.g., time, mental resources, money, etc.). When the body experiences stress, several physiological events occur driven by two branches of the Autonomic Nervous System, which is responsible for regulating bodily functions. The first branch is the Sympathetic Nervous System, which drives the body’s resources to respond to a challenge or a threat; sympathetic activation leads to an increased heart-rate and respiration rate, and tense muscles, a reaction that is known as the ‘fight-or-flight’ response [28]. During the fight-or-flight response, systems that are not essential to immediate survival, such as the digestive system, the reproductive system, and the immune system are suppressed. This process is complemented by the Parasympathetic Nervous System, which brings the body back to a state of relaxation. In non-stressful settings, these two systems work in coordination to achieve homeostasis, the condition where internal functions remain stable and balanced. In a stressful setting, the autonomic nervous systems are unbalanced. Prolonged imbalance in these two systems leads to long-term health problems. In the short term, acute stress is associated with negative feelings such as anger, frustration and fear [29], as well as lack of motivation, impaired decision making and decreased productivity [10,30].

The gold-standard in measuring stress is measuring the level of cortisol, known as the stress hormone. Cortisol measurements are used in clinical and lab studies (see [31] for a review), but they are unsuitable for workplace settings as they require collecting saliva or blood samples. As an alternative, continuous, unobtrusive stress tracking through other physiological measures has been explored.

In a survey of affective computing for stress detection, Greene et al. [32] list several measures of stress manifested in the human body that can be measured with existing technologies and that have been used in affective computing applications. They divide these bodily measures of stress into physical and physiological measures. Physical measures include facial expressions, eye activity, and body gestures, whereas physiological measures include cortisol level, brain activity, muscle activity, heart activity, skin response, respiratory activity and blood activity [32]. In this paper, we focus on physiological measures that can be obtained through unobtrusive wearable sensors.

### 2.2. Unobtrusive Physiological Sensing

For seamless and continuous stress monitoring in the workplace, stress measurement tools should not interfere with the user’s work or create additional stress and burden. Advances in wearable sensors and algorithms to analyze physiological signals enable continuous unobtrusive sensing of stress-related processes, which cannot be achieved with traditional self-report surveys. With recent wearable sensors, unobtrusive continuous monitoring of workplace stress is possible through capturing and analyzing physiological reactions to stress, such as changes in heart activity, skin conductance, and breathing. Sensors that capture these physiological signals can be embedded in comfortable wearable devices such as wristbands and chest bands. Besides wearable sensors, researchers have recently explored non-contact alternatives to approximate physiological signals. Digital cameras have been used to approximate physiological attributes based on analyzing facial expressions and subtle variations in skin tone (e.g., [33,34]). One of the most recent non-contact methods for unobtrusively measuring physiological signals associated with stress is thermal imaging. Thermal imaging provides a heatmap of the area of interest (usually the face) and highlights changes in surface temperature that are associated with stress, such as warming of the forehead [22] or perspiration in the perinasal area [24]. Thermal imaging for stress detection has been successfully validated in several contexts such as surgical training, security monitoring duty and office space [22,23,24,25,26].

### 2.3. Stress Monitoring in the Workplace: Literature Review

In this section, we review studies of stress monitoring in the workplace. The reviewed studies include research in workplace settings or computer use contexts. To narrow the scope of the review, we consider studies that use physiological signals to detect stress, and exclude studies focused on physical, facial and behavioral signals of stress (e.g., [35,36,37,38,39,40,41]). Studies approximating physiological measures with motion-based sensors such as accelerometers and gyroscopes (e.g., [42,43,44]) are also beyond the scope of this review.

Seventeen publications in areas spanning human–computer interaction, ubiquitous computing, biomedical informatics, user modeling, multimodal interaction, and affective computing from the years 2006–2017 were included in this review. Table 1, Table 2 and Table 3 summarize the reviewed studies based on the sensors and physiological signals, the computer task/stressor involved, the dependent variable (i.e., the stress measure), number of subjects, duration of physiological measurement and whether it is a lab or field study.

Most of the reviewed studies are controlled lab studies where subjects perform a task on the computer while wearing sensors to capture stress. The reviewed studies used computer tasks that simulate workplace computer use scenarios that might lead to stress. The tasks include computerized versions of validated stress-inducing tasks such as problem solving, solving puzzles, memory tasks, cognitive tasks, and mental arithmetic. Some tasks are validated stressors (such as the Stroop Color-Word test) while other tasks had additional stressors introduced (such as time pressure or social stress) to create the desired effect. For most studies, sample size ranged from 10 to 35 subjects, but varied in terms of unit of analysis (i.e., hours, sessions). A direct comparison of the results of all the above studies is not possible due to their differences in stress definitions, study design, sensors used, features extracted, and analysis methods.

The most common experimental setting in the reviewed studies was comparing a condition where stress was induced (e.g., by performing a stressful task or introducing social stressors), against another condition where no stress was induced. This approach results in binary classification models where data points are classified into either stress or rest. This classification is an oversimplification of workplace stress, as employees are seldom at rest (i.e., doing nothing). Some studies tried to address this limitation by increasing the number of classes (e.g., ‘relaxed’, ‘concentrated’, and ‘stressed’ in [50]) or replacing the ‘rest’ condition with non-stressful computer work (i.e., ‘low cognitive load’ vs. ‘stress’ in [51]). Other than predicting the stress condition, studies have also considered self-reports as ground truth, and used physiological signals as predictive variables (e.g., [20,49]). Finally, a stress measure that has been used, which captures more variation in stress, is departure from the baseline physiological measure, where stress is said to be detected if the physiological signal during the task is higher than the subject’s baseline measure (e.g., [21,22]).

While many studies measure stress during standardized computerized tasks (such as the Stroop Color-Word test) as a proxy for workplace computer use, Koldijk et al. [47,48] present a dataset of physiological measures during email interruption and time pressure as simulated workplace stressors, validated by self-reports of mental load. Using this dataset, Sriramprakash et al. [53] were able to build a model discriminating a neutral condition from the email interruption and time pressure condition using heart-rate and skin conductance measures. More work exploring workplace computer use scenarios beyond standardized computerized stressors is needed to account for the variation in workplace activities and the possible challenges for real-time stress monitoring during those activities, which is what we present in this study.

While the reviewed studies help advance unobtrusive stress measurement in the workplace, deploying these systems in real-life work scenarios requires a more nuanced understanding of the costs and benefits involved, and their robustness across different computer use scenarios in the workplace. We present a study where several sensor streams measuring stress were collected simultaneously during the performance of several computer tasks commonly performed at the workplace.

## 3. Methods

### 3.1. Experiment Design

**Figure 1:**
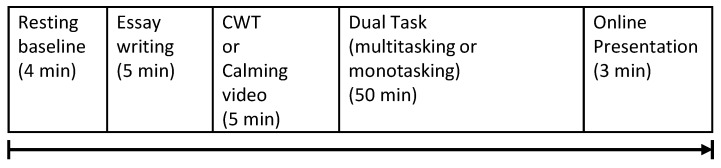
Experiment phases (CWT: Stroop Color-Word test).

As a part of a larger study on workplace stress, we simulated a workplace scenario where subjects conducted several tasks on the computer. The experiment consisted of several phases (Figure 1) starting with obtaining consent, filling out demographic and psychometric surveys, and equipment setup, followed by four minutes of rest to obtain baseline physiological measures for each subject. After the resting baseline period, subjects were asked to write an essay about a given topic for five minutes. Next, half of the subjects took the Stroop Color-Word test (CWT), while the other half watched a calm video. The Stroop Color-Word test is a validated stressor where the subject is shown a word designating a color, and the subject has to choose the color of the font of the word, rather than the color the word is designating. Next, subjects were asked to complete a 50-min dual task (DT) that consisted of writing an essay while responding to emails delivered under two conditions that represented high or low degrees of interruption. In the high interruption condition (multitasking), subjects were frequently interrupted by emails which they had to respond to as they arrived, simulating a multitasking computer work condition. In the low interruption condition (monotasking), subjects received the same number of emails but in a batch (i.e., all together) and had dedicated time to reply to them before returning to work on the essay. Finally, subjects were asked to present their essays in front of a virtual audience through video conferencing, a common real-life workplace task (In the original study, half of the participants were told in advance that they have to present in order to create anticipatory stress, while the other half were not told so. We found no difference in the physiological measures or self-reported stress of the two groups, hence, they are grouped together in this study.). More details about the experiment design, including additional measures collected but not used in this study, can be found in [8]. The experimental session, including preparation, setup and all tasks, lasted about 90 min. The experiment’s data, software systems, and data curation scripts along with the generated text and videos are publicly available at https://osf.io/zd2tn/.

The experiment was designed to minimize experimenter interaction to avoid additional stress caused by the experimenter’s presence. Software was designed so that each experimental phase could progress to the next, with minimal experimenter interaction. The experimenter sat in the same room and was separated from the subject by a partition. The experiment took place in a typical office booth with a desktop computer and a 24-inch display monitor. A webcam was placed on top of the monitor screen to video record the sessions so that it could be used to diagnose abnormal sensor readings.

### 3.2. Participants

Recruitment took place across three university campuses in the U.S. west and southwest through emails and flyers calling for participation. Participants had to be at least 18 years of age, have done all their schooling in English, and have at least a high school education to be eligible for this study. We recruited 96 participants, out of which 33 were excluded due to technical errors that caused their data not to be recorded. The remaining 63 participants (45 females and 18 males) were aged between 18 to 54 years, with a mean of 23.75 years (SD = 8.76 years). All participants signed informed consent and the study was approved by the institutional review boards of the participating universities. Two participants were removed as they had an abnormal resting baseline sensor reading. Two participants withdrew before or during the presentation session. Thus, data from 61 participants are included in this study, with the presentation session having 59 participants.

The majority (79%) of the participants were undergraduate students, 8% were graduate students, and 10% were employees. All participants rated themselves as fluent in English (score of 4+ on a 7-point scale) and the majority (93.4%) said they use email often in their daily work and life (score of 4+ on a 7-point scale), which makes them suitable for our study of computer work tasks involving writing essays and handling emails.

### 3.3. Sensors and Measures

We used well-validated sensors that provide measurements of the physiological changes that accompany stress. The following sensors and measures were used:

- Zephyr™BioHarness 3.0 Chest Strap with a BioModule: provides an ECG sensor for heart-rate (chest.HR) monitoring and an internal breathing sensor (BR). Subjects were instructed to wear the chest-band under their clothes for direct skin contact.
- Empatica E4 wristband: provides a PPG sensor for heart-rate monitoring (wrist.HR) and an EDA sensor. Participants wore the device on the wrist of their non-dominant hand.
- Thermal camera, Tau 640 longwave infrared (LWIR) camera (FLIR Commercial Systems, Goleta, CA, USA): Captures thermal images of subjects’ faces, from which perinasal perspiration (PP) is extracted using an algorithm by [24,58]. The thermal camera was placed under the participant’s computer monitor.

### 3.4. Data Normalization

There is no consensus in the literature on whether and how physiological features should be normalized by accounting for the baseline (i.e., tonic) or average level. Some studies show support for normalizing physiological features (e.g., [59]) while others found that models perform better with non-relative features (i.e., non-normalized) [51] or a mix of relative and non-relative features [60]. In our analysis we choose to normalize the obtained physiological signals by subtracting baseline values for each participant (i.e., physiological signals at rest) to account for individual differences and capture stress as a departure from the baseline level.

## 4. Results

### 4.1. Capturing Stress

We denote stress as a departure from the baseline physiological level for each participant. Equation (1) shows how stress was calculated for each participant (*P*), during each session (*S*), for each sensor stream (*E*). The stress in a given session is given by deducting the mean of physiological signal *i* during the resting baseline session for participant *j* from the mean of physiological signal *i* during session *k* for participant *j*, where *i* ∈{PP,BR,chest.HR,wrist.HR,EDA}, *k*∈{essaywriting,CWT,relaxingvideo,monotasking,multitasking,presentation}, *j*∈{1,…,63}.

(1) $$\Delta E_{i}\left(P_{j},S_{k}\right)=\bar{E_{i}}\left(P_{j},S_{k}\right)-\bar{E_{i}}\left(P_{j},S_{restingbaseline}\right)$$

We compare the set of sensor signals by their accuracy in capturing changes in stress during the different tasks by (1) using a t-test to determine whether there is a statistically significant difference between each session and the baseline; and (2) comparing the percentage of subjects for whom stress was captured by each sensor in each session.

#### 4.1.1. Essay Writing Session

During the first essay writing session, PP, chest.HR and BR showed a higher average than the baseline session (p0.001 for each). Signals from the wrist sensors (i.e., EDA and wrist.HR) did not show a statistically significant difference from baseline. Table 4 shows the ratio of subjects with a mean difference greater than 0 (i.e., higher than baseline) for the writing task. PP picked up the increased stress for 90% of the subjects, exceeding other signals which might not be as sensitive or generalizable across subjects. Since this task involves typing and moving the wrists, a potential explanation for the poor signal of EDA and wrist.HR for many subjects could be the influence of motion artifacts on the signal obtained from electrodes and PPG sensor on the wrist, and potential friction or detachment of the sensors which can cause sudden peaks or drops in the signal.

#### 4.1.2. Color-Word Test

This task is a validated stressor, and its computerized version is used in many simulated workplace stress studies (e.g., [52,55]) as a proxy for cognitively demanding computer tasks at the workplace. Ideally, all measures should show a significant increase in stress. However, in our data, only two measures detected a higher level of stress (Table 5). PP and BR showed a statistically significant difference from baseline (p0.01), chest.HR showed a trend of an increase (p0.06), while wrist sensors (EDA and wrist.HR) did not show a statistically significant difference. Although the overall EDA average across participants is not significantly higher than the baseline, EDA was higher during this session for 83% of the subjects, which might indicate that a few outliers affected the overall average. This task involved using the mouse with the dominant hand, so we do not expect that the wrist sensor placed on the non-dominant hand was affected by motion artifacts.

#### 4.1.3. Watching a Relaxing Video

We expected that watching a relaxing video would not generate any increase in stress compared to the baseline for any of the sensor streams. We found that PP captured a small increase in stress, while chest.HR captured a decrease in stress. The increase in PP was the smallest across all the sessions, which aligns with our expectation. Similarly, no other session generated as much decrease in chest.HR. These results for PP and chest.HR were to a large extent generalizable across subjects, with 79% of subjects showing a small increase in PP, and 76% of subjects showing a decrease in chest.HR. For other sensor streams, the difference from baseline was not statistically significant (Table 6).

#### 4.1.4. Dual Task—Monotasking and Multitasking

When subjects work on two tasks, whether monotasking or constantly switching between tasks, their stress level is expected to increase [61]. In our data, we found that PP and BR captured an overall statistically significant increase during the dual task (p0.001), while chest.HR, wrist.HR, and EDA did not. In terms of percentage of subjects showing an increase in stress, PP captured the increase for most subjects (Table 7 and Table 8). Given that this task includes typing, typically with both hands, we expect that wrist sensors had motion artifacts that might have affected the signal.

#### 4.1.5. Presenting to a Virtual Audience

Giving a presentation is a validated stressor in previous studies [3,4,62]. We expected that all sensor streams would show a significant increase in stress during this session. Our results (Table 9) show that only PP and chest.HR have an overall statistically significant increase in stress (p0.0001) with 96% and 82% of subjects showing higher stress than baseline level for PP and chest.HR, respectively. For both PP and chest.HR, the average increase in stress during the presentation session is the highest across all sessions, which is aligned with our expectations. For BR, we expect that the signal would be affected by speech respiratory patterns [63], which might explain the non-discriminant signal. Wrist sensors did not capture an overall statistically significant difference from baseline, although a higher stress level was captured for 74% and 53% of subjects for EDA and wrist.HR, respectively.

Overall, PP with the thermal camera detected an increase in stress for all computer tasks, with the highest increase being during the presentation session, and the lowest increase being during the video watching session, as expected. HR from the chest-worn sensor detected an increased stress only during essay writing and presentation sessions. BR showed an increase in all stressful sessions except presentation, where speech respiratory patterns interfere with the signal. Signals from the wrist-worn sensor (i.e., EDA and wrist.HR) did not capture increased stress for any session overall, although increased stress was captured for many individual subjects (50% of subjects in some sessions).

### 4.2. Sample Participant Data

Besides the statistical tests to determine whether stress was detected, we visually inspected each participant’s data for each sensor stream to identify patterns and potential issues. Below we provide examples of the patterns and issues identified, which explain and visualize the results of the previous section on capturing stress.

#### 4.2.1. EDA

**Figure 2:**
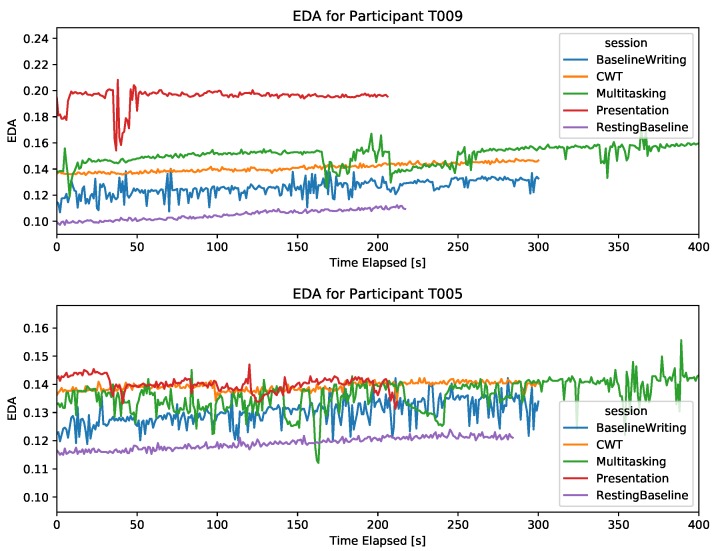
EDA signal during the five sessions for two participants. The *x*-axis is cut at 400 s, thus only showing the first 400 s of the DT.

For most subjects, the EDA level was close to zero, with small amplitude dynamics (i.e., small difference between the signal’s extreme values). Typically, a signal with small amplitude dynamics and no significant phasic activity (i.e., no abrupt peaks) makes it challenging to capture physiological changes associated with a task, especially in the absence of a discrete stimuli. However, we found that the tonic EDA level (i.e., the slowly increasing smooth pattern) differed among sessions for some participants. Figure 2 shows two examples where the baseline level is the lowest, and the presentation session has the highest EDA, as expected. It is important to note that the tonic EDA level can be naturally increasing over time, which contributed to finding significant differences among the sessions. However, as can be seen in the second chart in Figure 2, the order of the increase in EDA level does not always follow the chronological order of the sessions (i.e., the CWT session has higher tonic EDA than the DT, although CWT preceded DT). This figure also shows the effect of typing on the quality of the signal, as it shows more noise in the essay writing session and in certain bouts during the dual task session, compared to sessions that did not require typing. The severity of signal disturbance due to typing differed among subjects, with some subjects having significantly more noise than others.

For 18 participants, no significant signal was detected (i.e., EDA 0.02 for all sessions) and hence the stress level in different tasks could not be distinguished.

#### 4.2.2. Heart-Rate (Wrist PPG Sensor)

**Figure 3:**
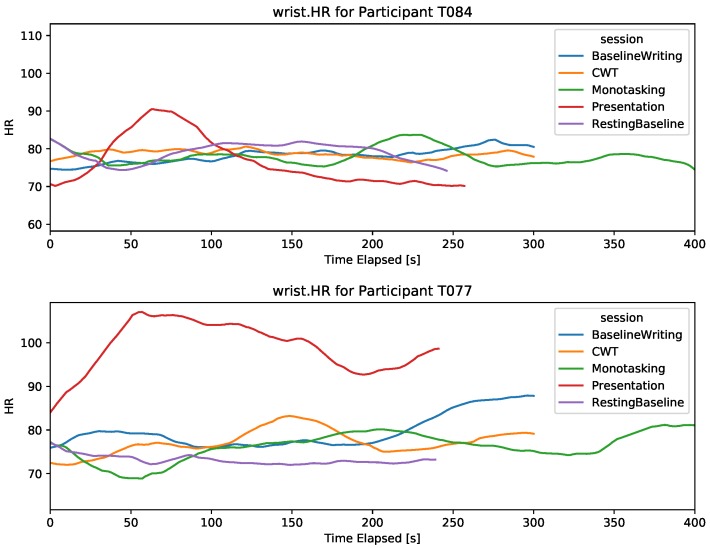
Wrist.HR signal during the five sessions for two participants. The *x*-axis is cut at 400 s, thus only showing the first 400 s of the DT.

The wrist-worn HR sensor provided the average heart-rate with a sampling frequency of 1 Hz. The HR signal is filtered by the device to remove motion artifacts. It is expected that HR increases with stress. For most participants, it could not be established that the resting baseline is the lowest HR across the different tasks, as can be seen in Figure 3. However, a higher HR was detected for some participants during the presentation session, suggesting that HR from a PPG wrist sensor can capture strong stress reactions but is non-discriminant for lower stress reactions. Thus, the wrist sensor did not provide an HR signal that can capture stress in different computer task scenarios.

#### 4.2.3. Breathing Rate

**Figure 4:**
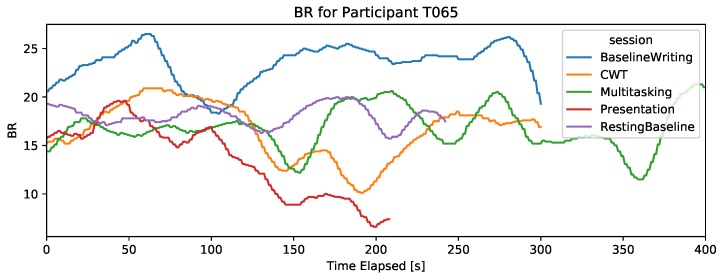
An example of a participant’s BR data showing degrading signal in the presentation session. The *x*-axis is cut at 400 s, thus only showing the first 400 s of the DT.

As can be seen in the examples in Figure 4, the breathing rate signal degrades in the presentation session, even if it shows a higher BR than baseline for some subjects.

#### 4.2.4. Heart-Rate (Chest ECG Sensor)

**Figure 5:**
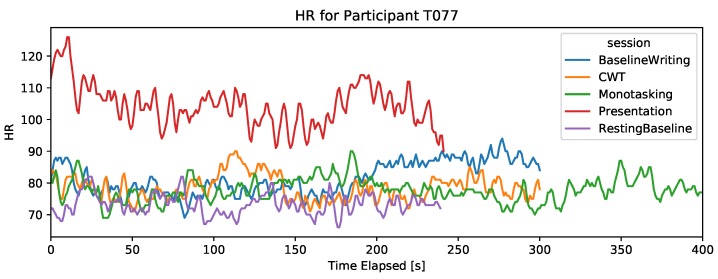
An example of a participant’s chest.HR data where increased stress is captured during stressful tasks.

**Figure 6:**
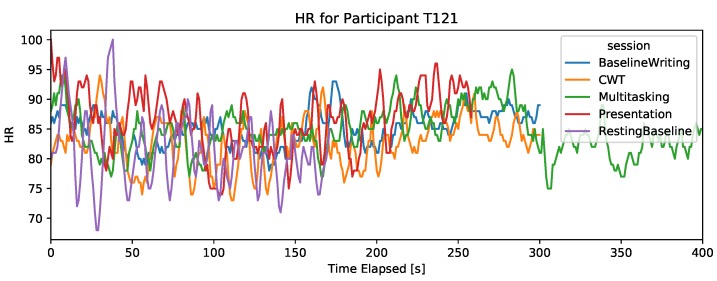
An example of a participant’s data with overlapping high frequency responses in the baseline session, likely due to sensor friction and detachment from the skin.

For most participants (83%), chest.HR detected the expected increased stress during the presentation session. Figure 5 shows an example of a participant with a clear difference in HR during different sessions. For participants where no significant difference was detected, inspecting the data showed some high frequency responses that made the signals from different sessions overlap (Figure 6), making capturing stress responses difficult.

#### 4.2.5. Perinasal Perspiration

**Figure 7:**
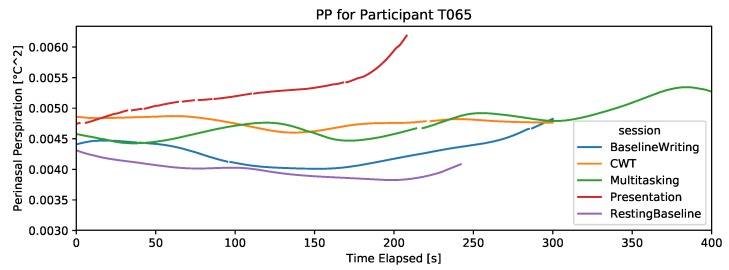
Example of a participant with PP signal that captures increased stress in stressful sessions. This participant took the color-word test and received emails in batches in DT (monotasking). This example also shows instances of missing data during the presentation session.

**Figure 8:**
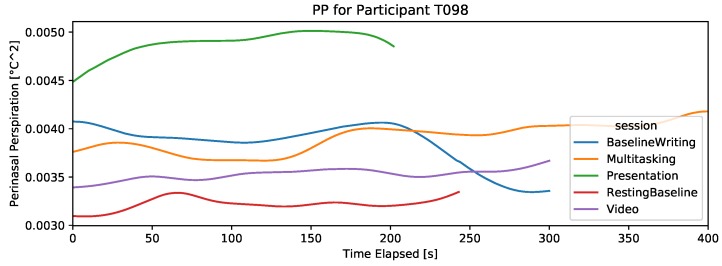
Example of a participant with the PP signal that captures increased stress in stressful sessions. This participant watched the relaxing video and received emails continually in DT (multitasking).

For most participants, the PP signal shows smooth patterns with clear distinctions among the different sessions. Figure 7 and Figure 8 show examples from a participant who received the color-word test as the third task, and another participant who received the relaxing video as the third task in the experiment, to eliminate the potential confound of potentially naturally increasing PP over time. As can be seen in Figure 7 and Figure 8, the relaxing video is the closest to the baseline PP level, while the color-word test is closest to the presentation PP level, as expected.

### 4.3. Missing Data

The thermal camera is the only non-wearable sensor in our experiment. While wearable sensors are attached to the skin and provide continuous readings, the thermal camera’s continuous reading is dependent on having the participant in a relatively still position facing the camera. Therefore, we investigate gaps in the recorded PP readings across different tasks to assess the suitability of using thermal imaging in different computer use contexts.

**Figure 9:**
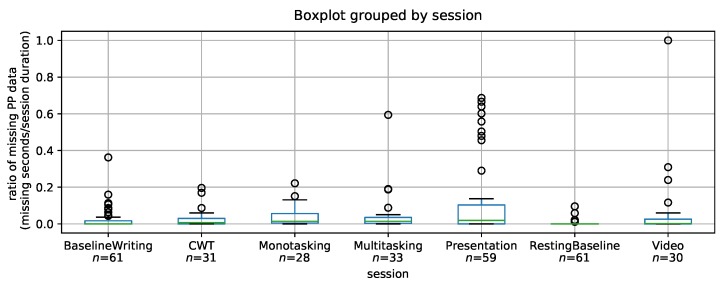
Boxplot of the ratios of missing PP data for all participants per session.

As can be seen in Figure 9 and Table 10, the thermal camera captured perinasal perspiration continuously with fewer gaps in tasks where subjects were sitting still, looking straight with minimal head movement. The session with the least missing data percentage is the resting baseline, with less than 10% missing data for each individual subject (average 0.3% for all subjects).

The virtual presentation session had the highest percentage of missing data, reaching more than 50% for some subjects. After revisiting the captured thermal video, we noticed that subjects were moving more than other sessions, which causes the perinasal area tracker to be lost. However, the average percentage of missing data is only 11%, with half the participants having less than 2% missing data. In a previous study by Hernandez [45], physiological measures obtained in an in situ study had an average of 0% missing data for wrist EDA, and 8%, 20% and 39% missing data for chest sensors’ HR, HRV, and BR, respectively. Our results with thermal imaging outperform chest sensors in terms of providing continuous readings and minimizing missing data. However, an in situ study spanning several workdays is needed to make a direct comparison with previous studies.

Some instances of missing data with thermal imaging were successfully avoided or recovered by having the experimenter re-select the perinasal area during the experiment, or by post-hoc analysis of the recorded thermal videos. Missing data also occurred when the experimenters failed to focus the camera on the participant at the beginning of the experiment, or anytime when the camera’s focus was lost during the experiment.

Overall, the average ratio of missing PP data is low (Table 10) with presentation being the session with the highest average ratio of missing data (11%) and the remaining sessions having between zero and 6% missing data, with at least 50% of subjects having less than 2% missing data in each session.

## 5. Discussion

Given the variety of computer tasks conducted at the workplace, our analysis showed that some sensors do not perform accurately to capture stress during certain tasks. For wrist-worn sensors, several reasons could cause the failure to capture stress. Sensor readings are prone to different types of sensor artifacts. For example, sensor electrodes can move, detach from the skin, or change in pressure on the skin, all which can affect the sensor signals, especially in dexterous tasks such as typing. In addition, we used a wrist sensor with dry electrodes, which depend on sweat for conductance. Thus, for calm sedentary users in an air-conditioned lab, the EDA signal might require a length of time of skin contact with the electrodes for the signal to appear. Previous studies have reported that detection of small EDA responses with wrist sensors is problematic [64], which might also explain why EDA did not detect the mild stress from computer tasks in our study. Palmar EDA (EDA obtained from the palm, or palm side of fingers) have shown better results for classifying calm and distress in sedentary settings in previous studies [65], but can be uncomfortable to wear during some computer activities. Finally, some subjects naturally do not produce adequate EDA signal in at least one wrist [66].

Many studies on unobtrusively capturing workplace stress with physiological sensors focus on specific high-stress computerized tasks. With a similar rationale as our study with common office tasks, McDuff et al. [34] considered more realistic everyday computer activities that require cognitive processing and dexterity. Their selected computer activities could introduce motion artifacts that can negatively influence the quality of the physiological readings and introduce physiological changes associated with body motions. Among other findings, McDuff et al. [34] report that HR and BR alone were not very discriminative indicators of cognitive stress, although their previous work showed BR to be significantly different during cognitive tasks compared to rest periods. Therefore, they suggest that BR might be dependent on the type of task and thus less generalizable. Another study with common office computer tasks (i.e., email interruptions) also reported low accuracy for predicting stress with HR and EDA [47]. Our findings are consistent with previous studies on common workplace computer tasks, showing that BR and HR for capturing stress are task-dependent.

While chest-worn sensors can provide an accurate reading for HR and BR, several considerations must be taken into account to ensure acquiring a good signal and reducing noise. For example, posture is important to avoid an abnormal signal. HR signals from the chest-worn sensor can drop to zero if the sensor disconnects due to crouching. HR signal can also be abnormally high due to sensor friction with the skin producing strong high frequency responses. Ramos et al. [60] reported that they instructed participants to refrain from leaning against the back of the chair to avoid signal noise introduced into the BR readings from the chest-worn sensor when the device was pressed against other objects, which makes wearing the sensor during real-life work contexts uncomfortable. Lastly, BR as a measure of stress is not accurate when the subject is talking, which restricts some workplace scenarios for using this signal to detect stress. These limitations introduce a usability problem with a cost-benefit tradeoff, where producing a good signal might require uncomfortable posture and restricted activities.

Additional filtering for noise reduction can partially address artifact-contaminated signals. However, since the focus of this study is to highlight the issues for different sensor streams during several common computer tasks, we did not pursue developing algorithms for further denoising. Previous work has investigated approaches to process artifact-contaminated data. For example, Hernandez [45] used a motion-sensor to detect ‘still’ moments in daily activities to opportunistically measure HR and respiration within the detected still motion time. Another approach by Alamudun et al. [67] suggest a preprocessing technique to remove the effects of factors interfering with physiological signals (e.g., posture or physical activity). They used a method called orthogonal signal correction, which attempts to remove any source of variance that is orthogonal to the dependent variable of stress level. Another method they used is linear discriminant correction, which models the source of noise (i.e., posture or physical activity) and removes it from the physiological signals. Their methods improved stress prediction from physiological data from an accuracy of 53.5% to 76.3%. However, it is unclear whether these approaches that have been developed for physical activities such as walking can successfully address motion artifacts from crouching or finer-grained activities such as typing.

Considering all methods, we found that perinasal perspiration with a thermal camera is the most generalizable method to capture stress across different tasks, as it can capture even slight changes and is robust against subject movement during computer tasks, providing reliable and continuous measurement with minimal missing data.

Our findings reveal that stress measurement in workplace environments, though important to do, is challenging, and relying on a single modality has many limitations. Previous studies have provided support for multimodal stress measurement given that physiological, personality, gender, sensor location and subject posture affect the selection of the best features to predict stress [8,45,50,68,69]. We extend those findings to show that the performed tasks also affect the choice of the best sensor signals. It may, however, be impractical to use multiple types of sensors. Therefore, thermal imaging appears to offer the most benefit in terms of usability and signal validity and reliability in the context of sedentary computer work.

In terms of usability, all sensors used in the study are unobtrusive and do not interfere with people’s ability to perform computer tasks. Sensors using electrodes (i.e., EDA and ECG sensors) can be uncomfortable for long-term use, as the electrodes become sticky after prolonged contact with the skin. This problem is avoided with non-contact thermal imaging. For data collection and analysis, all devices used come with software that collects and processes raw signals in real time, which is useful for human–computer interaction researchers who want to use these sensors in lab or in situ studies. Thermal imaging has the additional advantage of having the thermal video, which allows for revisiting the video to investigate abnormalities and re-extract features. In terms of cost, all devices have low costs during use and the main cost is the upfront cost of the device.

### 5.1. Scientific Contribution

The main contribution of our work lies in the breadth of sensor comparisons we used and the context in which they took place. A few other studies in affective computing have conducted sensor comparisons (e.g., [32,70,71]). However, our study is the first to compare thermal imaging and wearable sensors, capturing multiple physiological variables from different parts of the body with different measurement techniques. The breadth of sensors investigated positions this study as a reference for researchers and practitioners (see Section 5.3).

Another distinct and important contribution is the context of this study. Most previous studies of empirical comparisons of sensors take place in a context of either using a highly restricted experimental task in the laboratory (e.g., the Stroop Color-Word test) or as observations in the wild. In the case of restricted, standardized tasks conducted in the laboratory, experimenters have control over confounding factors, but ecological validity is compromised, which raises questions about the relevance of the findings for real-world applications. Sensor measurements done with an experimental task in an abstract lab environment may lack important characteristics that are associated with office tasks, such as time pressure or semantic context. In the case of field studies, ecological validity is high, but confounding factors are hard to control, which affect the robustness of sensor comparisons. The context of this current study aimed for ecological validity with multiple common computer tasks, instead of using an abstract laboratory task. Hence, while we controlled for confounding factors, the computer tasks we used are generalizable to real-world office tasks, which makes the sensor comparisons more relevant for use in the workplace. We used a variety of office tasks that were complex, and common in the information workplace, such as answering email and giving presentations.

Lastly, as a result of our sensor comparisons, our empirical study showed thermal imaging to be a robust stress measurement technique that is suitable for workplace and computer use settings, as it is less affected by confounding variables that introduce noise to other wearable sensor streams. Moreover, physiological sensing with thermal imaging has a capacity for correction, because it is not a one-dimensional temporal signal, but a derivative signal from imagery, which can be improved with better extraction processes or algorithms, even years after its original capture. This finding and the empirical testing of thermal imaging in a realistic context advances affective sensing methods and has implications for researchers and system builders.

### 5.2. Limitations

Our analysis investigated five common sensor streams. However, there are more physiological signals that can be unobtrusively monitored to measure stress that were not covered in our study. For example, heart-rate variability (HRV), blood volume pulse (BVP) and skin temperature (ST) can be extracted from sensors embedded in wearables [72]. Future work can compare HRV, BVP, and ST with other physiological signals during different workplace computer tasks.

Finally, despite having simulated a workplace environment which allowed us to investigate specific computer tasks, deploying sensors in real-life contexts can have additional challenges that cannot be modeled in lab settings. In the lab setting, careful instrumentation and real-time inspection of the sensor streams ensured high-quality signals. While our study discussed some challenges that are likely to occur in real-life settings, in situ studies can uncover additional validity and usability challenges for unobtrusive stress monitoring in the wild.

### 5.3. Insights for Researchers and System Builders

Our work has insights and implications for researchers and system builders, which could be synopsized as follows:

1. Controlled experiments are necessary to study cause and effect by isolating nuisance factors. This, however, does not imply that experimentation needs to be void of realism. In studies of stressful computer-based tasks, researchers relied for too long on standardized treatments alone, such as the Stroop Color-Word test (CWT), to investigate phenomena of interest. Such standardized treatments need to be accompanied by carefully designed realistic tasks (e.g., report writing interrupted by emails in the present study) if the goal is to generalize to real-world applications. Importantly, as the sensing results demonstrated in our study, the stress responses generated by standardized treatments often underestimate the stress responses generated by controlled realistic tasks, and thus potentially by real tasks in the wild as well.

2. All unobtrusive physiological sensors—wearable and imaging—are affected by motion artifacts. The advantage of imaging (thermal imaging in this case), however, is that the physiological signals are extracted algorithmically from video streams. Hence, one can visually identify the cause of noise (e.g., head turn) in the original source and compensate for it, either by removing the specific signal segment or by applying an algorithmic correction. In wearable sensor signals, this is more difficult, because there is no primary source of information (i.e., a 3D matrix) out of which these signals are extracted. The 1D temporal signal is all that the wearable sensor provides, and thus identification of motion artifacts is purely conjectural.

## 6. Conclusions

We have empirically compared five physiological signals that are known to be associated with stress. Across six computer tasks, perinasal perspiration captured through thermal imaging was the most generalizable as it captured even small changes in all tasks and for most participants. Heart-rate and breathing rate from chest-worn sensors captured changes in stress for some tasks, while heart-rate and EDA from wrist-worn sensors did not capture significant changes in stress, overall. We highlighted the effect of movement during typing tasks, and the effect of speaking during the presentation task. These findings advance our understanding of the complexity of computationally modeling workplace stress. With its breadth of sensor comparisons and realistic context, our study addressed a gap in the affective sensing literature. Our study is a step towards effective unobtrusive monitoring of stress in the workplace taking into consideration the various tasks and the challenges they introduce for stress monitoring.

## Figures and Tables

**Table 1:** Sensors and Signals of the reviewed studies.

Publication	Sensor: Signal
[20]	Wrist sensor: PPG, EDA, ST
[45]	Chest sensors: HR, HRV, BR; Wrist sensors: EDA, ST
[46]	Wrist sensor: EDA, ST, acceleration
[47]	Chest sensors: HR, HRV; Finger sensor: EDA; Cameras; Kinect 3D
[48]	Chest sensors: HR, HRV; Finger sensor: EDA; Cameras; Kinect 3D
[22]	Thermal imaging of the corrugator
[21]	PPG: sVRI, blood pressure; ECG: HRV
[34]	Digital camera: HR, BR and HRV
[49]	Smartphones: audio, physical activity, social interaction; Chest belts: HRV
[50]	Pressure sensor; eye-tracker; fingertip sensor: EDA, BVP, HR
[51]	Hand sensor: EDA
[52]	Necklace sensor: ECG; Fingertip sensor: EDA and ST; Chest sensor: BR.
[53]	Chest sensors: HR, HRV; Finger sensor: EDA
[54]	Chest belt: ECG and respiration; Hand sensor: EDA; Shoulder electrodes: sEMG
[55,56,57]	Hand sensor: BVP, EDA, ST; Eye-tracker: PD.
This work	Wristband: PPG (HR) and EDA; chest-band: ECG (HR), BR; Thermal camera: PP

Abbreviations: PPG: Photoplethysmogram, EDA: Electrodermal Activity, ST: Skin Temperature, HR: Heart-Rate, HRV: Heart-Rate Variability, BR: Breathing Rate, sVRI: Stress-Induced Vascular Response Index, ECG: Electrocardiogram, BVP: Blood Volume Pulse, sEMG: Surface Electromyogram, PD: Pupil Diameter, PP: Perinasal Perspiration.

**Table 2:** Computer tasks/Stressors of the reviewed studies.

Publication	Computer Task/Stressor
[20]	MIST
[45]	Unconstrained work environment
[46]	Unconstrained work environment
[47]	Writing reports with email interruptions and time pressure
[48]	Writing reports with email interruptions and time pressure
[22]	CWT and mental arithmetic
[21]	Arithmetic problems
[34]	Cognitive tasks: ball control task and BCST
[49]	Unconstrained environment—in and outside of work
[50]	CWT and information pick up task
[51]	MIST
[52]	CWT; talking about stressful experiences; math test
[53]	Writing reports with email interruptions and time pressure
[54]	Problem solving, puzzle, and memory task, done under time pressure, social pressure, and distracting noise
[55,56,57]	CWT
This work	CWT, relaxing video, multitasking, monotasking, essay writing, online presentation

Abbreviations: MIST: The Montreal Imaging Stress Task (mental arithmetic under time and evaluation pressure), CWT: Stroop Color-Word test, BCST: The Berg Card Sorting Task.

**Table 3:** Summary of reviewed studies.

Publication	Dependent/Output Variable	# Subjects	Duration of measurements	Controlled
[20]	STAI-Y	Lab: 21, Field: 5	Total: 1564 min (lab), 1327 h (field)	Partially
[45]	Self-report	15	5 days	No
[46]	EDA level	10	4 weeks	No
[47]	Self-report	25	3 h	Yes
[48]	Self-report	25	3 h	Yes
[22]	Difference from baseline	11	12 min	Yes
[21]	Physiological measures	40	50 min	Yes
[34]	Stress condition	10	10 min	Yes
[49]	Self-report	35	4 months	No
[50]	Stress condition	10	21 min	Yes
[51]	Stress condition	33	4 h	Yes
[52]	Stress condition	20	20 min	Yes
[53]	Stress condition	25	3 h	Yes
[54]	Stress condition	30	40 min	Yes
[55,56,57]	Stress condition	32	10 min	Yes
This work	Difference from baseline	61	90 min	Yes

Abbreviations: STAI: State-Trait Anxiety Inventory. Controlled: Whether data is collected in a controlled lab experiment.

**Table 4:** Results for the essay writing session. Delta: the difference between the session and the Resting Baseline.

Signal	Mean Delta	std	*t* (Delta ≠ 0)	*p* (Delta ≠ 0)	% of Delta 0
PP	0.000539	0.000714	5.899	0	90
chest.HR	2.641	4.977	3.789	0.001	78
BR	3.421	3.542	7.296	0	86
EDA	−0.006	0.597	−0.086	0.932	69
wrist.HR	−2.546	13.22	−1.171	0.249	51

**Table 5:** Results for the CWT session. Delta: the difference between the session and the Resting Baseline.

Signal	Mean Delta	std	*t* (Delta ≠ 0)	*p* (Delta ≠ 0)	% of Delta 0
PP	0.000432	0.000708	3.401	0.002	71
HR	2.192401	5.536	1.98	0.059	68
BR	2.724293	3.127	4.692	0.001	79
EDA	0.098085	0.379	1.098	0.287	83
wrist.HR	0.361225	6.09	0.252	0.804	50

**Table 6:** Results for the video session. Delta: the difference between the session and the Resting Baseline.

Signal	Mean Delta	std	*t* (Delta ≠ 0)	*p* (Delta ≠ 0)	% of Delta 0
PP	0.000404	0.000937	2.322	0.028	79
HR	−2.703	5.304	−2.548	0.018	24
BR	0.557	2.991	0.985	0.333	54
EDA	−0.14	0.534	−1.115	0.281	72
wrist.HR	−1.389	9.002	−0.673	0.51	32

**Table 7:** Results for the monotasking session. Delta: the difference between the session and the Resting Baseline.

Signal	Mean Delta	std	*t* (Delta ≠ 0)	*p* (Delta ≠ 0)	% of Delta 0
PP	0.000777	0.000882	4.657	0.001	79
chest.HR	−0.994	4.567	−1.11	0.278	35
BR	1.19	2.3	2.534	0.019	79
EDA	−0.017	0.133	−0.545	0.593	67
wrist.HR	−3.087	9.877	−1.326	0.202	33

**Table 8:** Results for the multitasking session. Delta: the difference between the session and the Resting Baseline.

Signal	Mean Delta	std	*t* (Delta ≠ 0)	*p* (Delta ≠ 0)	% of Delta 0
PP	0.001	0.001	5.603	0	85
chest.HR	−0.55	7.934	−0.347	0.732	52
BR	2.357	4.448	2.902	0.007	67
EDA	−0.606	2.62	−0.982	0.34	78
wrist.HR	−2.5	11.756	−0.927	0.366	47

**Table 9:** Results for the presentation session. Delta: the difference between the session and the Resting Baseline.

Signal	Mean Delta	std	*t* (Delta ≠ 0)	*p* (Delta ≠ 0)	% of Delta 0
PP	0.002146	0.001476	11.167	0	97
chest.HR	9.832	12.487	5.34	0	83
BR	−0.57	4.226	−1	0.322	44
EDA	−0.484	2.329	−1.228	0.228	74
wrist.HR	1.479	13.604	0.652	0.518	53

**Table 10:** Mean and median of the ratio of missing PP data per session.

Session	Mean	Median
Essay writing	0.024	0.000
CWT	0.027	0.007
Presentation	0.109	0.019
Resting Baseline	0.003	0.000
Monotasking	0.036	0.014
Multitasking	0.046	0.014
Calming Video	0.064	0.002

## Abbreviations

The following abbreviations are used in this manuscript:

BCST	The Berg Card Sorting Task
BR	Breathing Rate
BVP	Blood Volume Pulse
CWT	Stroop Color-Word test
DT	Dual Task (email and essay)
ECG	Electrocardiogram
EDA	Electrodermal Activity
HR	Heart-Rate
HRV	Heart-Rate Variability
MIST	The Montreal Imaging Stress Task
PD	Pupil Diameter
PP	Perinasal Perspiration
PPG	Photoplethysmogram
sEMG	Surface Electromyogram
ST	Skin Temperature
STAI	State-Trait Anxiety Inventory
sVRI	Stress-Induced Vascular Response Index
